# The US Chinese Anti-Cancer Association and the Asian Fund for Cancer Research Recognize Young Chinese Cancer Researchers with the 2016 USCACA–AFCR scholar awards

**DOI:** 10.1186/s40880-017-0197-4

**Published:** 2017-03-17

**Authors:** Wei Zhang, Li Yan, Wei Zhang, Yunguang Tong, Shi-Yuan Cheng

**Affiliations:** 1 US Chinese Anti-Cancer Association, Los Angeles, Martinez, CA 94553 USA; 2 Department of Preventive Medicine, Northwestern University Feinberg School of Medicine, Chicago, IL 60611 USA; 3 Beijing Cancer Hospital and Institute, Peking University School of Oncology, Beijing, 100142 China; 4 Department of Cancer Biology, Wake Forest University, Winston-Salem, NC 27157 USA; 5 Department of Medicine, Cedars-Sinai Medical Center, Los Angeles, USA; 6 University of California, Los Angeles, School of Medicine, Los Angeles, CA USA; 7 Department of Neurology, Northwestern Feinberg School of Medicine, Chicago, IL 60611 USA

To foster and strengthen collaborations among cancer researchers and physicians in the United States (US) and China, the US Chinese Anti-Cancer Association (USCACA), the National Foundation for Cancer Research (NFCR), and the Asian Fund for Cancer Research (AFCR) have established the USCACA–NFCR/AFCR Scholarship Program in Basic, Translational, and Clinical Studies. Between 2010 and 2015, 24 junior Chinese cancer researchers and physicians have been recognized by this award for their outstanding achievements in cancer research accomplished both during their training in the US and after their returning to China [1–4]. In 2016, three young researchers were selected on the basis of their significant contributions in translational cancer research. The USCACA and AFCR proudly presented the Award to the following three outstanding young scientists during the 5th Guangzhou International Symposium on Oncology held in Guangzhou, Guangdong, China, December 1–3, 2016 (Fig. 1).

**Fig. 1:**
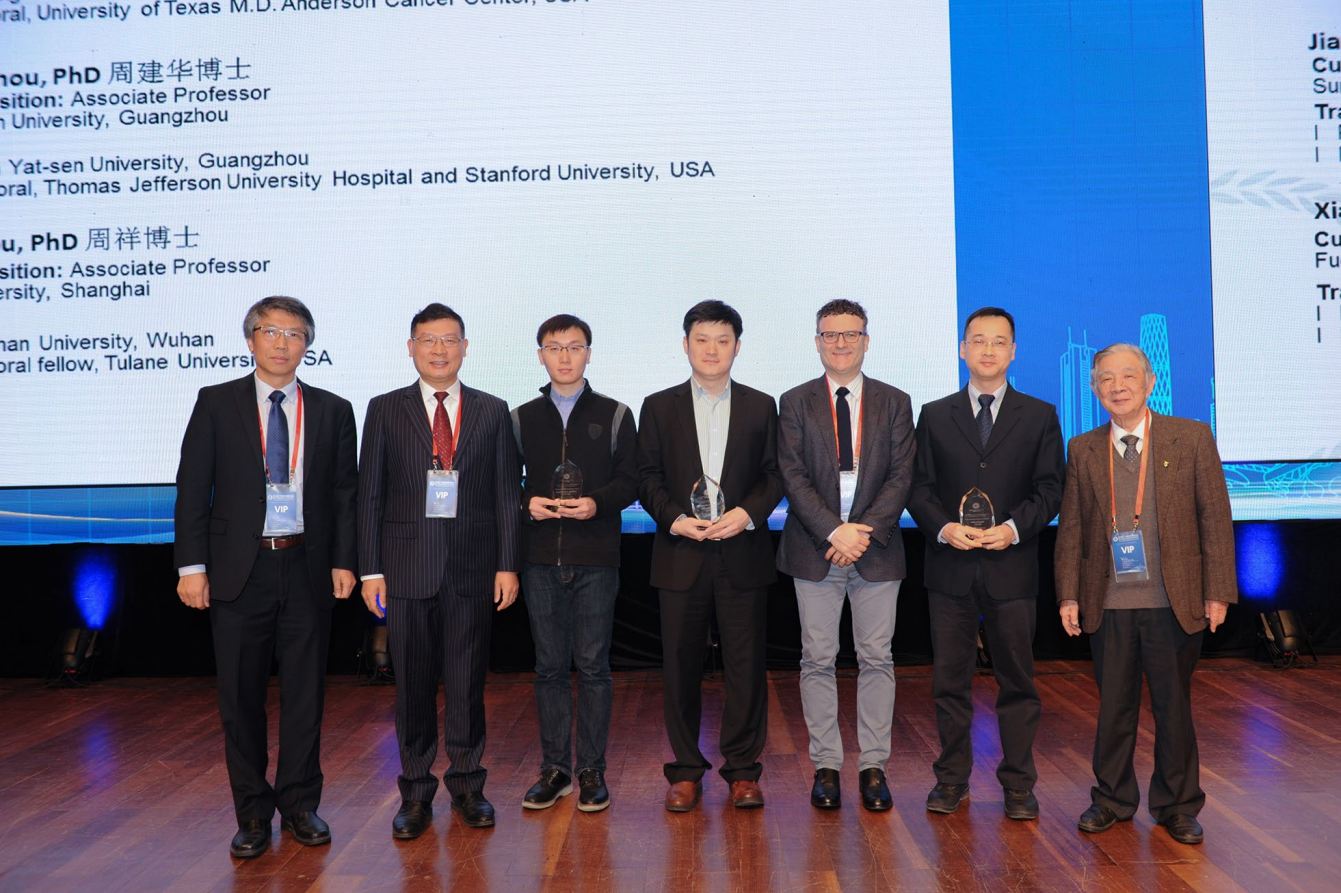
The ceremony of the 6th USCACA–AFCR Scholar Awards was held in Guangzhou on December 2, 2016. Awards were presented by Dr. Wei Zhang from the USCACA Executive Committee. From *left* to *right* Dr. Wei Zhang (Wake Forest University), Dr. Ruihua Xu (the President of Sun Yat-sen University Cancer Center), Dr. Xiang Zhou, Dr. Song Gao, Dr. Boris Pasche (Director of Wake Forest Baptist Comprehensive Cancer Center), Dr. Jianhua Zhou, and Dr. Zhongzhen Guan (a former president of Sun Yat-sen University Cancer Center). USCACA, the US Chinese Anti-Cancer Association; AFCR, the Asian Fund for Cancer Research

- Dr. Song Gao, Department of Pancreatic Cancer, Tianjin Medical University Cancer Institute and Hospital, Tianjin, China
- Dr. Jianhua Zhou, Department of Ultrasound, Sun Yat-sen University Cancer Center, Guangzhou, Guangdong, China
- Dr. Xiang Zhou, Cancer Institute in Shanghai Cancer Center, Fudan University & Institute of Biomedical Sciences, Shanghai, China

The three winners of the 2016 USCACA–AFCR Scholar Awards were invited to an award ceremony at the opening ceremony of the 5th Guangzhou International Symposium on Oncology on December 2, 2016 in Guangzhou, China. All awardees have received excellent doctoral and/or postdoctoral training under their US mentors who are leading cancer researchers and USCACA members. The discoveries and findings from these talented young scientists have not only significantly enhanced our understanding of the complex mechanisms underlying the development and progression of human cancers but also provided critical insights for identifying novel targets and developing new approaches for improving treatment and clinical care of cancer patients.

## The 2016 USCACA–AFCR scholars

Dr. Song Gao is currently an attending physician in the Department of Pancreatic Cancer at Tianjin Medical University Cancer Institute & Hospital. He received his Ph.D. degree in oncology in 2015 from Tianjin Medical University. Dr. Gao became a faculty at Tianjin Medical University Cancer Institute & Hospital in 2009. He joined Professor Wei Zhang’s laboratory in the Department of Pathology at the M.D. Anderson Cancer Center, University of Texas, Houston, TX, USA as an exchange Ph.D. student in 2013. During his PhD studies, Dr. Gao demonstrated that insulin-like growth factor-binding protein 2 (IGFBP2) is a potential oncogene that contributes to malignant behaviors of pancreatic ductal adenocarcinoma. The exciting results were reported in *Cancer Research*. Currently, Dr. Gao is focused on mutual regulation of hypoxia-inducible factor-1 (HIF-1) and IGFBP2 in exosome secretion from pancreatic cancer cells. As an oncology surgeon specializing in pancreatic cancer, he continues to work with his colleagues on an IGFBP2 project in pancreatic cancer as well as being an active member of the surgery team. Dr. Gao has been a key research investigator on two research projects sponsored by the National Natural Science Foundation of China.

Dr. Jianhua Zhou joined Prof. Juergen K. Willmann’s laboratory in the Department of Radiology, Stanford University, Stanford, California, USA in November 2014. During his tenure at Stanford, he investigated whether three-dimensional ultrasound molecular imaging (3D USMI) of vascular endothelial growth factor receptor 2 (VEGFR2)/kinase insert domain receptor (KDR) expression could accurately gauge longitudinal treatment responses to anti-angiogenic therapy in responding versus non-responding colon cancer mouse models. Dr. Zhou demonstrated that tumors in these models exhibited differential patterns of VEGFR2-targeted 3D USMI signals during anti-angiogenic therapy. In responding tumors, the VEGFR2 signals decreased as soon as 24 h after initiation of therapy, whereas in non-responding tumors there was no change in signals at any time point. The early decrease in VEGFR2 signals was highly predictive of treatment outcome at the end of therapy. His findings suggest that 3D USMI could be further developed for monitoring early treatment changes in cancer patients. This work was published in *Cancer Research* in 2016. In December 2015, Dr. Zhou returned to Sun Yat-sen University Cancer Center to continue his career as an associate professor. His current research has been funded by three grants from the National Natural Science Foundation of China. The goal of his investigations is to provide noninvasive approaches for early monitoring tumor response to chemotherapy in clinic.

Dr. Xiang Zhou is currently an associate professor at Shanghai Cancer Center and Institutes of Biomedical Sciences of Fudan University. He received his BS degree in biotechnology in 2004 and Ph.D. degree in genetics in 2009 from Wuhan University, Wuhan, Hubei, China. In 2009, Dr. Zhou joined Dr. Hua Lu’s laboratory at Indiana University School of Medicine, Indianapolis, Indiana, USA as a postdoctoral fellow and, later on, he moved to Tulane University School of Medicine, New Orleans, Louisiana, USA with Dr. Lu as a research scientist. Dr. Zhou’s research was to understand the mechanisms underlying the regulation of p53 and c-Myc. One of his research projects was to elucidate the mechanism of ribosomal (nucleolar) stress-mediated tumor suppression. Dr. Zhou demonstrated that, in response to ribosomal stress, several ribosomal proteins can activate p53 or p73 by inhibiting negation of mouse double minute 2 (MDM2) and inactivate c-Myc through their direct interaction as well as microRNA pathways. These studies were separately published in *Oncogene*, *Cell Death & Differentiation*, and *Journal of Biological Chemistry*. In addition, Dr. Zhou and his colleagues identified two p53-inducible genes, *NGFR* and *PHLDB3*, which promoted chemoresistance by suppressing p53 activity as feedback regulators. These results were published in *Elife* and *Nature Communications*, respectively. Dr. Zhou’s current research interests focus on the translational study of ribosomal stress-induced tumor suppression and dissection of the oncogenic mechanism of mutant p53 during tumorigenesis.

## About US Chinese Anti-Cancer Association (USCACA)

USCACA is a non-profit professional organization founded in 2009 (http://www.uscaca.org/). With members from academia, industry, and government, USCACA facilitates collaborations among cancer researchers and physicians in the US and China. We currently focus on expediting novel cancer drug development by fostering clinical trial networks, sharing expert medical practices and knowledge of clinical trials, and providing education and training opportunities. USCACA collaborates with Chinese Anti-Cancer Association (CACA), Chinese Society for Clinical Oncology (CSCO), Chinese Medical Association (CMA), and Chinese Society for Oncology (CSO), as well as other professional associations. Our mandate is to improve cancer treatment through research, education, and collaboration.

## About the Asian Fund for Cancer Research (AFCR)

The AFCR is a non-profit organization committed to curing cancers that have significant impacts on Asian populations. Headquartered in Hong Kong, AFCR is uniquely positioned to implement in Asia the newest cancer research discoveries and technologies around the world, investigate the distinct causes of cancer in Asian populations through innovative genetic and molecular researches, and develop more effective therapies tailored to Asian cancer patients. AFCR is dedicated to bridging the scientific and educational gaps in cancer research and cancer prevention between Asian countries and the rest of the world through promoting, coordinating, and funding international collaborations in cancer research and public education.

## The common goal of USCACA and AFCR

The common goal of USCACA and AFCR is to conquer cancer by improving our understanding of cancer and providing more efficacious and safe treatment options to cancer patients through expediting novel cancer drug development that stimulates the translation of laboratory discoveries into novel cancer treatments. We aim to encourage the translation of laboratory discoveries into novel therapeutic treatments of cancer patients, foster collaborations in scientific discovery, cancer drug development, and clinical treatments, and share expert knowledge and medical practices between China and the US. The USCACA–AFCR Scholarship Program provides a unique opportunity for junior Chinese scholars who have an interest in advancing their basic, translational, and clinical knowledge and skills in cancer research. It also allows these young scholars to establish long-term collaborations with leading cancer researchers in the US who can support their continued work and future success in China.
